# Patient With Prior COVID-19 Infection Presenting With Acute Upper Airway Obstruction: A Case Report

**DOI:** 10.7759/cureus.23214

**Published:** 2022-03-16

**Authors:** Claudia Sorin, Sugi Min, Gerardo Carino

**Affiliations:** 1 Pulmonary and Critical Care, Brown University, Providence, USA; 2 Internal Medicine, Columbia University Irving Medical Center, New York City, USA

**Keywords:** covid-19, acute airway obstruction, pharyngitis, post-covid sequelae, epiglottitis

## Abstract

New clinical manifestations of coronavirus disease 2019 (COVID-19) have been emerging throughout the pandemic and are being reported to the medical community. There have been limited reports that the virus can cause acute airway compromise. Here, we describe a young patient with a recent COVID-19 infection now presenting with acute airway compromise, presumed to be pharyngitis, and their clinical course during their hospitalization. The purpose of this case presentation was to shed light on a newly reported, presumed, presentation of COVID-19 that can be life-threatening in people of all ages. Though there have been limited reported cases, it is important to include this virus in the differential diagnosis of virus-induced airway compromise.

## Introduction

The novel coronavirus disease 2019 (COVID-19) can cause a rapidly progressive respiratory illness and can cause complications involving other major organs [1]. Limited reports of acute airway compromise, including tonsillitis or epiglottitis, among patients, both adults and children, with COVID-19 have been described and suggest that these may be rare, late complications among COVID-19 patients [2,3]. Acute airway obstruction is a life-threatening event requiring emergent identification and management. Here, we describe a young patient with a recent COVID-19 infection presenting with acute airway compromise, presumed to be pharyngitis, and his clinical course during his hospitalization.

## Case presentation

**Figure 1:**
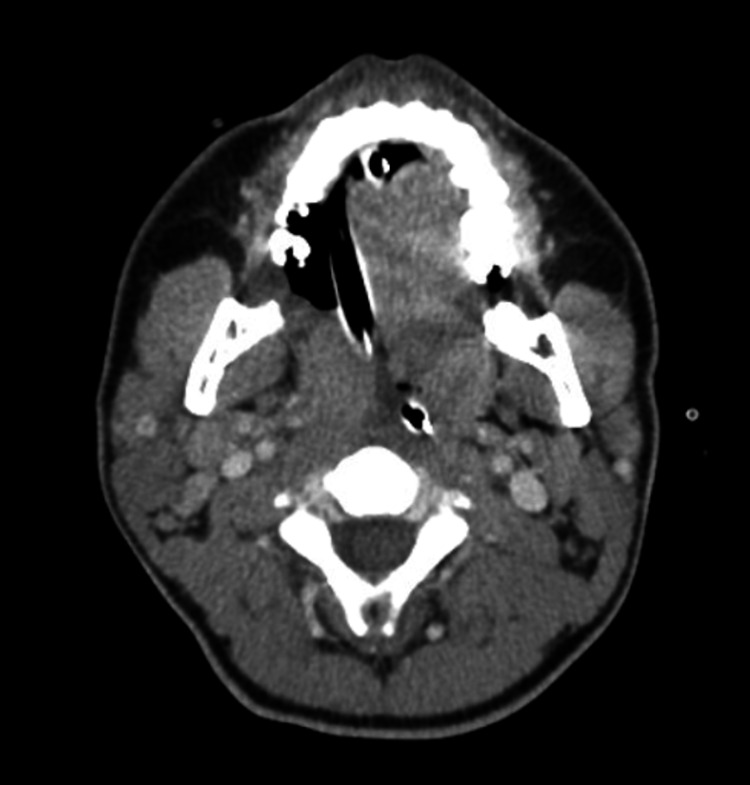
Axial view of patient’s upper airway post-intubation with diffuse hypertrophy of the tonsillar tissue, adenoids and lingual tonsils, extensive edema around endotracheal tube.

**Figure 2:**
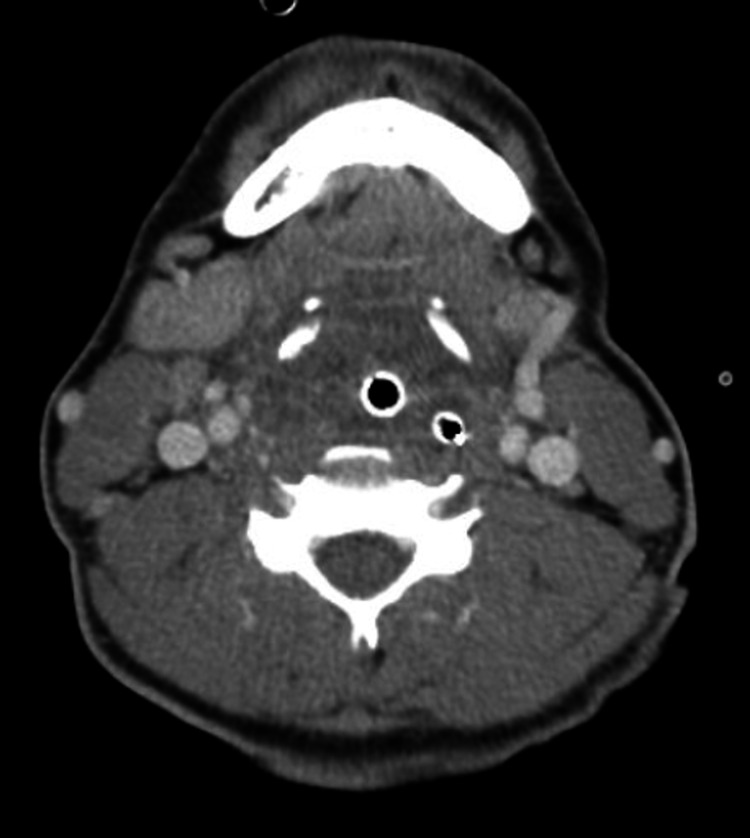
Axial view of patient’s upper airway post-intubation with diffuse hypertrophy of the tonsillar tissue, adenoids and lingual tonsils, extensive edema around endotracheal tube.

**Figure 3:**
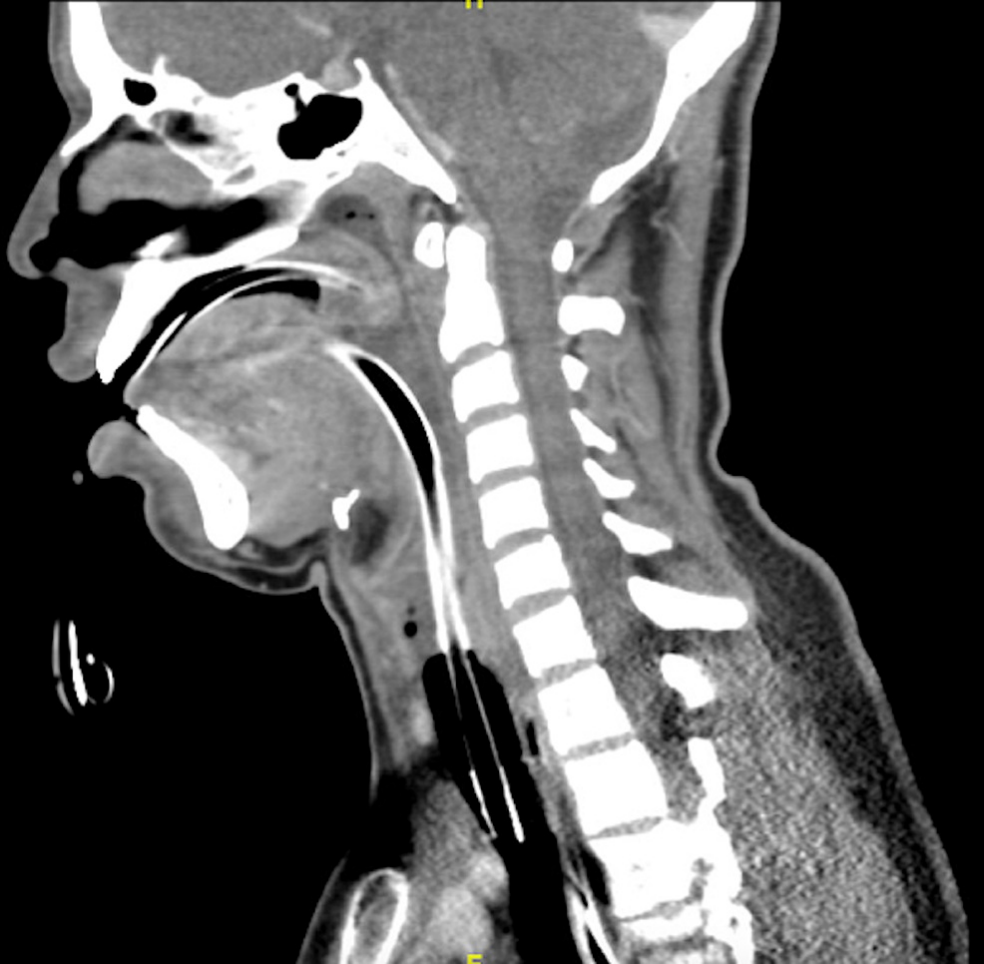
Sagittal view of upper airway post-intubation.

A 19-year-old Guatemalan male with no known past medical history, unclear vaccination status (for COVID-19 or other transmissible diseases), non-smoker, no alcohol use, presented to the emergency department in December 2020 with complaints of worsening sore throat, muffled voice, difficulty swallowing secretions, bloody vomitus, and subjective fevers. He was diagnosed with mild COVID-19 via reverse-transcriptase-polymerase-chain-reaction assay (RT-PCR) three weeks prior. History was limited by severe respiratory distress. On physical examination, the patient was afebrile but ill-appearing and diaphoretic. Vitals were notable for tachypnea with a respiratory rate between 22 and 26 breaths per minute while on a non-rebreather mask with an oxygen saturation of 100%, tachycardia of 141 beats per minute, and blood pressure was 188/95. Other significant physical examination findings included muffled voice and stridor. The uvula was visible without edema or deviation. Labs were significant for positive severe acute respiratory syndrome coronavirus 2 (SARS-CoV-2) antigen via RT-PCR with the remainder of the respiratory pathogen panel negative, and leukocytosis to 19,600, 85% of which were segmented neutrophils and 1% were bands. The patient was given an initial dose of dexamethasone and empiric ampicillin-sulbactam. X-ray of chest and neck were both unremarkable. Given concern for airway compromise and possible epiglottitis, the patient was urgently intubated; the first attempt with video laryngoscopy was unsuccessful due to extensive edema and erythema of the hypopharyngeal tissues obscuring the vocal cords, then re-attempted successfully with a smaller endotracheal tube. The epiglottis could not be directly visualized. CT of the neck showed diffuse inflammatory changes with enlargement of the adenoids and tonsils (Figures 1-3). The epiglottis was obscured given the presence of the endotracheal tube. No abscess was noted. 

The patient was admitted to the intensive care unit for further management and was continued on dexamethasone 10 mg with later change in dosage to 6 mg every six hours for two days, and eventually tapered off on hospital day five, and empiric ampicillin-sulbactam, which was later narrowed to cephalexin on hospital day four. ENT and infectious disease services were both consulted. Differential diagnoses included bacterial or viral epiglottitis, idiopathic angioedema reaction, or post-COVID reaction. Ebstein-Barr virus (EBV) IgM and IgG, heterophile antibody, bacterial tests (rapid strep test, mycoplasma pneumoniae and chlamydia pneumoniae), and HIV antibody/antigen were unremarkable. Cultures of larynx obtained by ENT showed light growth of methicillin-sensitive *Staphylococcus aureus* (MSSA), deemed to reflect colonization. Viral polymerase chain reaction (PCR) tests of nasopharynx and epiglottis were both positive for SARS-CoV-2. The patient had rapid resolution of airway edema following a 40-hour course of dexamethasone and broad-spectrum antibiotics and was successfully extubated on day four of admission. He was discharged home on hospital day six after clinical improvement with a 10-day course of cephalexin. 

## Discussion

This study highlights a young patient with a recent COVID-19 infection presenting with acute upper airway compromise requiring emergent endotracheal intubation, managed as presumptive epiglottitis. Acute epiglottitis should be suspected in adult patients presenting with odynophagia, fever, muffled voice, drooling, stridor, and/or hoarseness. Stridor is the most important finding in respiratory obstruction [4]. Diagnosis is typically confirmed visualization of an erythematous and edematous epiglottis by visualization during direct or indirect laryngoscopy, or on oropharyngeal examination. The patient described did not have confirmed epiglottitis but was treated as such given their physical examination findings and their acute upper airway obstruction. Direct visualization would have been helpful in confirming the diagnosis given the lack of imaging findings to suggest epiglottitis. Lateral radiographs can provide diagnosis, with the classic “thumb sign,” but radiographs were abnormal in only 77-88% in two larger adult case series. In cases of acute respiratory distress, as presented in this case, due to the risk of rapid respiratory compromise airway management should supersede diagnostic evaluation [5,6]. 

Notably, this case presented late three weeks after the initial diagnosis of COVID-19. Given most mild, symptomatic cases of COVID-19 are known to resolve within two to three weeks, these late presentations raise concern for epiglottitis as a rare but serious complication of COVID-19. Additionally, the time course suggests possible post-viral superinfection of the epiglottis and upper airway from other viral or bacterial causes. Research by Wölfel et al. has found the presence of separate genotypes of SARS-CoV-2 in throat swabs and sputum that support that there is independent viral replication in the throat [7]. This raises the question of a delayed inflammatory condition isolated to the epiglottis or subglottic structures leading to severe airway compromise.

While dyspnea and pharyngitis are common presenting symptoms in COVID-19, the incidence of epiglottitis and subsequent airway compromise among patients with COVID-19 is not known. Survey of the literature revealed two case reports of acute epiglottitis in adult patients with confirmed COVID-19 infection; one involved near-complete airway obstruction due to marked epiglottic enlargement, seen on computed tomography of the neck, requiring emergent cricothyrotomy [8], and another had edematous epiglottis seen on flexible bronchoscopy and required ICU admission but no intubation was reported [2]. This case had clinical resolution of symptoms within one week of initial presentation. Separately, a case series detailed laryngotracheitis involving significant ulceration and edema of the epiglottis, glottis, and subglottis in two patients mechanically ventilated with COVID-19 [9]. These changes were present despite the resolution of clinical and radiographic characteristics of COVID-19 and improvement based on reduced oxygen and ventilation requirements. It is not clear whether these cases of laryngotracheitis were a late and prolonged response to COVID-19 of this part of the airway, or a complication of endotracheal intubation itself, and thus should be investigated further.

## Conclusions

There are many known etiologies of acute airway obstruction, including epiglottitis, but it is important to consider COVID-19 as another possible etiology. While direct visualization is the gold standard of confirming diagnosis in cases of epiglottitis, diagnosis should not precede emergent intubation in cases of severe respiratory distress. Treatment remains the same as standard of care - steroids and empiric antibiotics for possible secondary bacterial infection. Sharing this new information of this COVID-19 manifestation among subspecialties, including otolaryngologists and anesthesiologists, can aid in diagnosing and treating these patients accordingly.
